# Evaluation of the limitations and methods to improve rapid phage-based detection of viable *Mycobacterium avium* subsp. *paratuberculosis* in the blood of experimentally infected cattle

**DOI:** 10.1186/s12917-016-0728-2

**Published:** 2016-06-16

**Authors:** Benjamin M. C. Swift, Jonathan N. Huxley, Karren M. Plain, Douglas J. Begg, Kumudika de Silva, Auriol C. Purdie, Richard J. Whittington, Catherine E. D. Rees

**Affiliations:** School of Veterinary Medicine and Science, Sutton Bonington Campus, Loughborough, Leics LE12 5RD UK; School of Biosciences, University of Nottingham, Sutton Bonington Campus, Loughborough, Leics LE12 5RD UK; The University of Sydney, Farm Animal and Veterinary Public Health, Faculty of Veterinary Science, Camden, Australia

**Keywords:** Johne’s disease, Paratuberculosis, Mycobacteria, Bacteriophage, Bacteraemia, Blood test, Detection method

## Abstract

**Background:**

Disseminated infection and bacteraemia is an underreported and under-researched aspect of Johne’s disease. This is mainly due to the time it takes for *Mycobacterium avium* subsp. *paratuberculosis* (MAP) to grow and lack of sensitivity of culture. Viable MAP cells can be detected in the blood of cattle suffering from Johne’s disease within 48 h using peptide-mediated magnetic separation (PMMS) followed by bacteriophage amplification. The aim of this study was to demonstrate the first detection of MAP in the blood of experimentally exposed cattle using the PMMS-bacteriophage assay and to compare these results with the immune response of the animal based on serum ELISA and shedding of MAP by faecal culture.

**Results:**

Using the PMMS-phage assay, seven out of the 19 (37 %) MAP-exposed animals that were tested were positive for viable MAP cells although very low numbers of MAP were detected. Two of these animals were positive by faecal culture and one was positive by serum ELISA. There was no correlation between PMMS-phage assay results and the faecal and serum ELISA results. None of the control animals (10) were positive for MAP using any of the four detection methods. Investigations carried out into the efficiency of the assay; found that the PMMS step was the limiting factor reducing the sensitivity of the phage assay. A modified method using the phage assay directly on isolated peripheral blood mononuclear cells (without PMMS) was found to be superior to the PMMS isolation step.

**Conclusions:**

This proof of concept study has shown that viable MAP cells are present in the blood of MAP-exposed cattle prior to the onset of clinical signs. Although only one time point was tested, the ability to detect viable MAP in the blood of subclinically infected animals by the rapid phage-based method has the potential to increase the understanding of the pathogenesis of Johne’s disease progression by warranting further research on the presence of MAP in blood.

## Background

*Mycobacterium avium* subsp. *paratuberculosis* (MAP) is a slow-growing bacterium that causes Johne’s disease, a wasting disease in ruminants and other animals. A common test for Johne’s disease is the serum antibody ELISA test which monitors the humoral immune response of the animal following MAP exposure. However there are well known limitations of this test, for example the sensitivity can be extremely low, especially during subclinical stages of infection [1]. It has been established that there can be a bacteraemic phase in paratuberculosis, which has been demonstrated by both PCR [2] and culture [3, 4]. However the PCR cannot differentiate between live and dead cells and the sensitivity of the PCR assay is limited by inhibitory substances in the blood and the likelihood of there being a low number of MAP cells present [5]. However, studies that have compared the detection of MAP in blood by PCR to ELISAs have found an association but poor correlations between the tests [5, 6]. The long periods of time required for culture of this organism (even using automated systems) means that to date very few studies of blood samples have been completed so that the incidence, intensity and duration of mycobacteraemia is not known in any animal species.

We have previously described the use of a rapid, bacteriophage-based method (phage amplification assay) coupled with PMMS to specifically detect and identify viable MAP cells in the blood of naturally infected animals [7]. The organism was detected in milk and serum ELISA positive animals, but not from a certified Johne’s disease free herd that was milk and serum ELISA negative. The PMMS-phage method was employed here as components present in the whole blood inhibited the phage assay to such as extent that the sensitivity of the assay was not useful [7]. Using PMMS it was possible the capture the MAP cells and suspend them in a medium suitable for the phage assay. The stages of natural infection are difficult to ascertain in naturally infected cattle and given the known limitations of the ELISA tests for diagnosis of early infection this was not surprising, but it means that more data is needed to understand the relationship between bacterial load during mycobacteraemia and the blood ELISA results. The aim of this investigation was to apply the PMMS-phage assay to determine whether MAP could be detected in the blood of experimentally exposed cattle, where their stage of infection could be defined. Previously we have demonstrated that the MAP cells are found within the PMBC fraction of blood [7], and we have defined this as bacteraemia even though the MAP cells are intracellular rather than free in the blood stream. It is also possible that the assay would also detect MAP cells free in the bloodstream but to date we have not formally tested this possibility. The presence of viable MAP in cattle blood has previously been demonstrated in serum ELISA positive, inconclusive and negative animals by the PMMS-phage assay in an uncontrolled environment [7], thus the significance of mycobacteraemia is unknown, when compared to tests that are insensitive when used on subclincally infected animals, such as the ELISA test. As it can be assumed that the presence of this organism in the blood of the animal indicates that it has crossed the gut and disseminated, the results would be used to determine if evidence of disseminated infection correlates with the recorded immune response of an animal in a controlled environment.

## Results

### Use of the blood assay on MAP-exposed, subclinically infected cattle

During our study, none of the animals had weight loss or signs related to Johne’s disease. Blood samples were collected at 4 years, 8 months post-exposure from all animals for the PMMS-phage assay. Faecal and blood samples were collected in the same month for faecal culture, PCR and serum antibody ELISA testing. The unexposed cohort remained MAP-negative as determined by faecal culture, serum Ab ELISA and faecal PCR (Table 1) by the end of the experiments. One of the exposed animals (#14) was euthanized due to reasons unrelated to MAP status. Samples from two of the control animals (#3 and #10) produced plaques, however none of these gave a positive result when the IS*900* PCR was performed, indicating that these plaques represented either breakthrough (incomplete inactivation of all extracellular phage particles results in *M. smegmatis* plaques) or detection of a mycobacterium other than MAP [8]. Therefore the phage results agreed with all other tests performed in the negative control herd. Thus none of the control animals were positive for MAP by any of the methods used.

**Table 1:** Results from the PMMS-phage assay, faecal culture, faecal PCR and serum ELISA for sub-clinical, experimentally exposed cattle to MAP

			Results			
Sample	Breed	MAP exposure status^a^	PMMS-Phage assay^b^	Faecal culture	Faecal PCR^c^	Serum Ab ELISA (%)^d^
1	Holstein	Control	- (0)	-	-	1.00
2	Holstein	Control	- (0)	-	-	29.34
3	Holstein	Control	- (2)	-	-	3.85
4	Holstein	Control	- (0)	-	-	5.92
5	Holstein	Control	- (0)	-	-	5.42
6	Holstein	Control	- (0)	-	-	3.14
7	Holstein	Control	- (0)	-	-	17.49
8	Holstein	Control	- (0)	-	-	10.49
9	Red/Holstein	Control	- (0)	-	-	3.50
10	Red/Holstein	Control	- (3)	-	-	4.57
11	Holstein	Inoculated	- (0)	-	-	2.57
12	Holstein	Inoculated	**+** (2)	-	*	3.28
13	Holstein	Inoculated	- (0)	-	-	5.00
14	Holstein	Inoculated	#^e^	#^e^	#^e^	#^e^
15	Holstein	Inoculated	- (5)	-	-	14.49
16	Holstein	Inoculated	**+** (5)	-	-	3.28
17	Holstein	Inoculated	- (0)	**+**	**+**	48.68
18	Holstein	Inoculated	**+** (2)	-	-	8.57
19	Holstein	Inoculated	- (0)	-	-	6.21
20	Holstein	Inoculated	**+** (2)	-	-	35.62
21	Holstein	Inoculated	- (2)	-	-	12.63
22	Holstein	Inoculated	- (0)	-	-	3.50
23	Holstein	Inoculated	- (0)	**+**	**+**	118.77
24	Holstein	Inoculated	- (0)	-	-	6.50
25	Holstein	Inoculated	- (0)	-	*	4.64
26	Red/Holstein	Inoculated	**+** (4)	-	-	5.21
27	Holstein	Inoculated	**+** (5)	-	*	41.11
28	Holstein	Inoculated	- (0)	-	-	2.00
29	Red/Holstein	Inoculated	**+** (3)	-	-	13.28
30	Red/Holstein	Inoculated	- (0)	-	*	1.50

^a^Inoculated – animals experimentally exposed to MAP; Control – animals not

exposed to MAP

^b^+/- indicates result of combined PMMS-phage –PCR assay. Plaque numbers

for each sample given in brackets

^c^* indicates MAP DNA was detected but the quantity detected was below the cut-point for a positive result [16]

^d^Serum Ab ELISA (IDEXX); Positive value > 55 %, suspected value 45–55 %

^e^# Animal 14 was culled due to other illness unrelated to Johne’s disease before sample collection

Two of the inoculated animals (#17 and 23) were shedding MAP in their faeces, according to the results of both faecal culture and faecal PCR. Animal #23 was also positive by serum Ab ELISA and animal #17 gave a borderline positive serum Ab ELISA (49 %; cut-off value for a positive test result = 55 %). Using the phage-PCR MAP assay, seven out of the 19 (37 %) MAP-exposed animals that were tested had positive results, indicating that viable MAP cells were detected in their blood samples. However compared to other studies we have performed, only very low numbers of plaques (2–5) were produced in the MAP-positive samples. Interestingly animals #20 and #27, which were positive for the presence of MAP in their blood according to the PMMS-phage assay, had the next two highest (36 % and 41 %, respectively, although classed as negative) serum ELISA test results (Table 1). However there was no overall correlation between the results of phage-PCR and either faecal PCR or serum Ab ELISA.

### Investigation of PMMS MAP isolation efficiency

As the number of MAP cells detected using the PMMS-phage assay was much lower than recorded in our previous studies [7], the efficiency of the PMMS isolation step was investigated. Cattle strains of MAP were 10-fold serially diluted from 1 × 10^4^ to 1 × 10^0^ viable MAP cells. These cells were inoculated in 7H9 medium and the PMMS method to isolate the MAP cells was carried out. The phage enumeration assay was then used to determine how many MAP cells were captured from the samples. It was found that for this batch of peptide-coated beads the limit of detection of the MAP cells was only 7.3 × 10^2^ pfu.ml^−1^ (Table 2). This indicated that the PMMS MAP isolation method was only isolating roughly 10 % of the MAP cells present in the samples, thus explaining the low levels of plaques recorded in in the blood of MAP-positive samples.

**Table 2:** Efficiency of phage-PMMS method in isolating and detecting MAP cells compared to MPN

Number of MAP Cells			
MPN	Number of Plaques		
10^4^	TNTC	TNTC	TNTC
10^3^	88	52	79
10^2^	0	5	4
10^1^	0	0	0
10^0^	0	0	0

*MPN* most probable number method for determining number of MAP cells [22]

*TNTC* too numerous to count

### Improving the detection of MAP by applying the phage assay to PBMCs isolated from blood

**Fig. 1:**
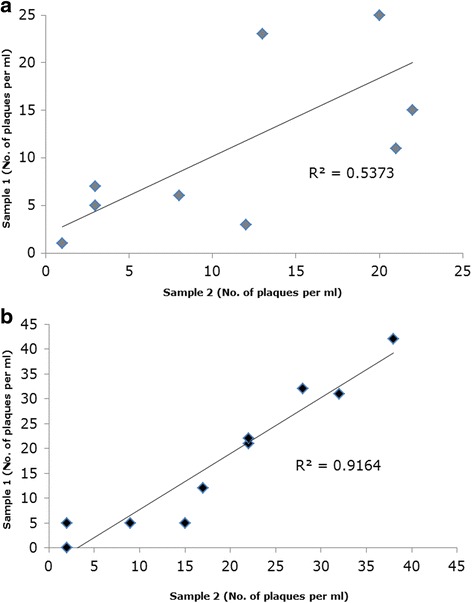
Reproducibility of detecting MAP from whole blood using PMMS and from PBMCs. Figure show the reproducibility of detecting viable MAP cells using paired samples tested independently with the phage assay using the PMMS to isolate the MAP cells from whole blood (**a**) or detecting the MAP cells with the phage assays directly from PBMCs isolated from sheep blood (**b**)

Since the PMMS step requires expensive reagents and optimisation of bead coating, experiments were designed to determine whether MAP cells could be detected from whole blood without using PMMS. It has previously been shown that MAP cells are present in the PBMC’s of the blood and not found in the plasma or red blood cell fractions [7]. Initially, simple limit of detection experiments were performed to determine whether the PBMCs inhibited the phage assay. MAP cells were enumerated using the method described by [9] and artificially spiked into modified 7H9 medium and the PBMCs isolated from sheep blood. The phage assay was carried out on the artificially inoculated PBMCs and modified 7H9 medium containing known numbers of MAP cells. The results show that there was no significant difference (*p* < 0.05) in the number of MAP cells detected by the phage assay in the control samples (containing only modified 7H9 medium) and in the PBMCs. Duplicate blood samples tested with the PMMS-phage assay and phage assay without PMMS on PBMCs. The paired samples tested independently with the phage assay, the detection of MAP in PBMCs was more reproducible (*R*^2^ = 0.92; Fig. 1b) than isolating MAP cells using the PMMS method on whole blood samples and detecting with the phage assay (*R*^2^ = 0.54; Fig. 1a).

## Discussion

Cattle infected with MAP may not show clinical signs of disease until years after exposure. Subclinically infected animals may shed MAP into the environment in their faeces and MAP cells can also be present in their milk, but both milk and blood ELISA testing on subclinical animals is notoriously insensitive [10, 11]. It is not known whether all animals that give a positive ELISA test result will go on to develop clinical Johne’s disease. The results of this study show the first application of the phage assay on experimentally infected animals and its comparison between faecal shedding and blood ELISA status. The results have shown that animals with variable blood ELISA status can have detectable levels of viable MAP in their blood. More data are required to better understand the relationship between the host immune response following MAP-exposure and disease progression, however having a clearly defined experimentally controlled study such as this is the first step in determining the usefulness of the phage assay. The ability to be able to detect MAP cells in blood samples from animals and obtain results within days (rather than within months), may aid the understanding of disseminated infection with regards to immune responses during disease progression and development of clinical disease and when this moves beyond a localised gut infection to a systemic phase.

It is also important to consider the type of bacteraemia detected in this study. As demonstrated here and previously, MAP can be detected in the PBMC fraction of blood and not others [7], so it is assumed that the MAP cells detected are internalised within PBMCs. In our experiments to establish the internalisation model, free MAP cells did not co-purify with the PBMCs supporting this model. The possibility exists however that bacteraemic animals have free MAP cells present in their blood that were not detected by our methods.

There were a number of differences in experimental design compared to a previous study in which we found viable MAP cells in whole blood ranging from 3–39 pfu.ml^−1^ [7]. The assay used in the present study was transferred from University of Nottingham laboratory, where the assay was initially established and optimised, to the University of Sydney. The availability of different equipment and the use of different reagents (particularly the peptide-coated beads) meant that some aspects of the method were not fully optimised before the trial was carried out. Furthermore, the animals in this study were castrated males and therefore were not exposed to the same reproductive and lactation stress as the milking dairy cattle used in the previous study [12]. These factors may explain the differences in the plaque number results obtained between the two studies.

The PMMS method requires expensive reagents, such as biotinylated peptides and paramagnetic beads and the use of PMMS introduced more variability into the results gained as tested with paired samples. Using the well-established Ficoll-Paque method to separate PBMCs from whole blood removes the need for PMMS and means that the phage assay can be carried out directly, reducing losses during sample processing. However this does reduce the selectivity of the method and therefore other mycobacteria potentially present in the blood may also be detected by the phage assay. This means that there is a greater reliance on the performance of the end-point PCR identification step to confirm the identity of the cell detected by the phage. Although limited by the inefficiency of the PMMS, the results showed that the assay was specific; despite plaques being detected in two of the unexposed animals, no MAP DNA was detected in these samples, which probably represents some phage break-through in the assay. This has been noted before and emphasises the importance of combining the phage assay with a subsequent PCR-identification step.

In the group of exposed cattle, exposure to MAP occurred over 4.5 years before blood samples were taken for testing and none of the cattle were showing clinical signs of Johne’s disease. Approximately one third of MAP-exposed cattle had viable MAP in their blood that was detectable by the PMMS-phage assay. Although two animals were shedding MAP in their faeces, no MAP was detected in the blood of these individuals. This was interesting as it would have been assumed, if the disease had progressed to the stage of detectable level of shedding, that the animals would be more likely to have viable MAP in their blood. However the results suggested that this was not necessarily the case. In subclinically infected animals especially, faecal shedding is intermittent. This study examined a single time-point and only small volumes of blood were tested. It is also important to note that bacteraemia is a transient event that can happen during any stage of infection, whether that is in the early subclinical stage of infection or later [13]. Thus, these findings may indicate that the presence of viable MAP cells in the blood may also be intermittent relative to when the animals are found to be shedding MAP in their faeces or may be a rare event or may be the different forms MAP are found in the blood may relate to different pathophysiological manifestations that may or may not be related to the lack of correlation between faecal shedding and dissemination into the blood stream. Thus testing additional aliquots from a single time point may assist in determining the true likelihood of detecting viable MAP in the blood.

## Conclusions

This is the first study to show that viable MAP may be present in the blood of experimentally MAP-exposed cattle prior to the onset of clinical signs and does not appear to be correlated to other measures, including faecal shedding and serum antibody responses, which are routine diagnostics used for Johne’s. The ability to detect viable MAP in the blood of subclinically infected animals may lead to increased sensitivity of diagnosis in the early stages of infection, but this requires validation. Here we have demonstrated the proof of concept in a controlled study where variations such as exposure (time and dose etc.) and environmental factors are limited [14]. Having a tool like the phage assay allows new questions to be asked possibly in a longitudinal study testing the blood, milk and faeces of animals to enable a better understanding of paratuberculosis disease progression, determine when systemic infection occurs in animals, and how this influences the serum and milk ELISAs, and faecal culture or PCR results.

## Methods

### Bacterial strains, bacteriophage and growth medium

To optimise MAP detection in the peripheral blood mononuclear cells (PBMCs), MAP strains K10 and ATCC 19698 were used. The *Mycobacterium smegmatis* strain was mc^2^155, which is used routinely in phage assays [15] and the bacteriophage used was D29. All liquid cultures of MAP were prepared in modified 7H9/OADC medium (Becton Dickenson, UK) supplemented with Mycobactin J (2 μg μl-1; Synbiotics Corporation, France) and when performing the phage assay the medium was supplemented with 2 mM CaCl_2_. MAP strain; CM00/416 was used to both establish the phage assay method in the University of Sydney laboratories and to infect the cattle. MAP strains used for experimental infection trials and for the investigation of peptide mediated magnetic separation (PMMS) isolation efficiency were grown as previously described by Plain [16] and enumerated using the most probable number method (MPN) as described by [17].

### Experimentally infected animals and sampling

All animal experiments carried out for this study were approved by The University of Sydney, Australia, animal ethics committee. An existing MAP infection trial being undertaken at the University of Sydney was used to provide samples for analysis (manuscript in preparation). In this trial 30 calves (aged 2–4 months) were age matched then randomly allocated into a group of 20 to be experimentally exposed to MAP (Numbers; 11–30, Table 1) along with a group of 10 age-matched unexposed control animals (Numbers; 1–10, Table 1). With consent, male Holstein and Holstein/Australian Red cross calves were selected from a property in New South Wales that was unexposed to MAP; there was no previous history of any MAP infection on the farm and both the dams and calves were confirmed to be MAP-negative by faecal culture and faecal PCR. The MAP exposure time points were determined using a previous study [18]. Calves at 3-4 months of age were experimentally inoculated orally with a cattle (C) strain of MAP (CM00/416/C4) using a protocol based on a validated ovine model [19]. The inoculation was carried out in three doses over a period of one month with the total dose of viable MAP administered being 9.46 × 10^9^ MAP cells. The control unexposed group consisted of 10 age-matched calves, and exposed and unexposed cohorts were maintained in different paddocks at the University of Sydney Camden farms to prevent cross-contamination. Control animals were maintained on paddocks where no MAP-infected livestock had been housed in the past. Faecal MAP culture was performed as previously described [20]. All samples tested and presented in this study were obtained from the animals 4 years, 8 months post exposure to allow the potential for MAP infection to establish. Faecal PCR was performed using a validated method as previously described [16] and serum antibody ELISA and results were expressed as signal of the test sample as a proportion of the positive control, corrected for the negative control (S/P %) calculated as described by the manufacturer (IDEXX Laboratories, Maine, USA).

### Peptide mediated magnetic MAP isolation and phage detection from blood

MAP cells were isolated from blood and detected using the phage assay using PMMS according to the method described by [7]. Briefly, magnetic beads were coated with peptides aMp3 and aMptD that specifically bind MAP [21]. Coated beads were mixed with 1 ml of whole blood diluted in 9 ml of modified 7H9 medium (10 ml total volume). Samples were mixed for 30 min to allow MAP to bind the beads before they were recovered by centrifugation (4500 × *g;* 15 min) and washed with 9 ml of modified 7H9 medium before recovered with centrifugation before being resuspended in 1 ml of modified 7H9 medium to carry out the magnetic separation. Beads were then concentrated by magnetic separation and resuspended in 1 ml of modified 7H9 medium prior to testing using the phage amplification assay.

The phage assay and experimental controls were carried out according to [15]. Briefly, 1 ml samples were mixed with D29 bacteriophage (100 μl; 1 × 10^9^ pfu.ml^−1^) in modified 7H9 Media and incubated for 1 h to allow the phage infection. Any remaining free phage were inactivated using 10 mM (final concentration) ferrous ammonium sulphate (6 min at room temperature whilst being continuously mixed). The ferrous ammonium sulphate was then inactivated with 5 ml of modified 7H9 medium before the samples were mixed with *M. smegmatis* cells (1 ml; 1 × 10^8^ pfu.ml^−1^) before plating with soft 7H10 agar (0.75 % agar). The number of MAP cells detected was determined by counting the number of plaques formed (data reported as pfu.ml^−1^) in the lawn of *M. smegmatis* cells. Enumeration of MAP cells in initial inocula was performed by diluting samples until countable numbers of plaques are obtained [9].

### PCR identification of MAP cells

After the phage assay, DNA from up to five plaques formed on each plate was extracted using a gel DNA extraction kit (ZymoResearch, UK). Then the nested IS*900* PCR assay was used to confirm that MAP DNA was present as described previously by [7]. The MAP specific IS*900* quantitative PCR was carried out using the method described by [16].

### Peripheral blood mononuclear cells isolation

The isolation of the PBMCs from cattle blood was carried out using Ficoll-Paque Plus according to the manufacturer’s instructions (GE Healthcare Life Sciences, UK). Briefly 2 ml of whole heparinised blood was mixed with 2 ml of PBS (Dulbecco A). This was carefully layered on 3 ml of Ficoll-Paque Plus in 15 ml falcon tubes. The samples were centrifuged (400 × *g,* for 30 min at 18 ^o^C). After centrifugation the upper layer (plasma) of the sample was removed. The buffy coat layer was carefully removed ensuring the red blood cells were not disturbed. The PBMCs were washed with 6 ml of PBS by centrifugation (100 × *g* for 10 min at 18 ^o^C). The supernatant was removed and the pellet was resuspended in 1 ml of modified 7H9 medium for the phage assay.

## Abbreviations

MAP, *Mycobacterium avium* subsp. *paratuberculosis*; MPN, most probable number; PMMS, peptide-mediated magnetic separation
